# Bis[benzyl *N*′-(3-phenyl­prop-2-enyl­idene)hydrazinecarbodithio­ato-κ^2^ *N*′,*S*]copper(II)

**DOI:** 10.1107/S1600536808002262

**Published:** 2008-01-25

**Authors:** M. T. H. Tarafder, M. Toihidul Islam, M. A. A. A. A. Islam, Suchada Chantrapromma, Hoong-Kun Fun

**Affiliations:** a Department of Chemistry, Rajshahi University, Rajshahi 6205, Bangladesh; b Department of Chemistry, Rajshahi University of Engineering and Technology, Rajshahi 6205, Bangladesh; c Department of Chemistry, Faculty of Science, Prince of Songkla University, Hat-Yai, Songkhla 90112, Thailand; d X-ray Crystallography Unit, School of Physics, Universiti Sains Malaysia, 11800 USM, Penang, Malaysia

## Abstract

The Cu^II^ atom of the title complex, [Cu(C_17_H_15_N_2_S_2_)_2_], lies on a twofold rotation axis, and is in a distorted tetra­hedral geometry with the two bidentate N_2_S_2_ Schiff bases. In the crystal structure, the mol­ecules are inter­connected into chains along the *c* axis by weak C—H⋯S inter­molecular inter­actions. The crystal packing is further stabilized by C—H⋯π inter­actions.

## Related literature

For bond-length data, see: Allen *et al.* (1987 ▶). For the synthesis and structures of *S*-benzyl­dithio­carbaza­tes, see: Ali & Tarafder (1977 ▶); Shanmuga Sundara Raj *et al.* (2000 ▶). For related Cu^II^ complexes, see: Ali *et al.* (2008 ▶); Castiñeiras *et al.* (1998 ▶); Goswami & Eichhorn (2000 ▶). For bioactivities of *S*-benzyl­dithio­carbazate metal complexes, see: Ali *et al.* (2002 ▶, 2008 ▶); Tarafder *et al.* (2001 ▶, 2002 ▶).
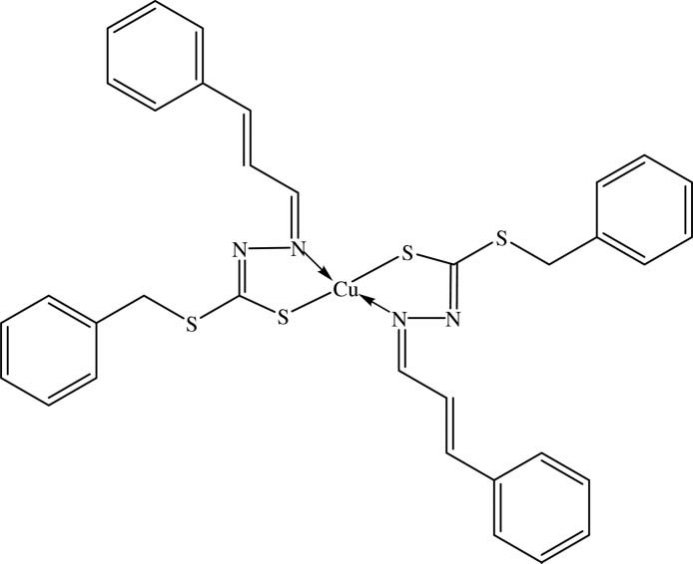


## Experimental

### 

#### Crystal data

[Cu(C_17_H_15_N_2_S_2_)_2_]
*M* *_r_* = 686.45
Orthorhombic, 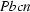

*a* = 36.1410 (7) Å
*b* = 9.9372 (2) Å
*c* = 8.7598 (2) Å
*V* = 3146.00 (11) Å^3^
*Z* = 4
Mo *K*α radiation
μ = 0.99 mm^−1^
*T* = 100.0 (1) K
0.57 × 0.29 × 0.10 mm

#### Data collection

Bruker SMART APEXII CCD area-detector diffractometer
Absorption correction: multi-scan (*SADABS*; Bruker, 2005 ▶) *T*_min_ = 0.603, *T*_max_ = 0.906
84850 measured reflections
6922 independent reflections
5675 reflections with *I* > 2σ(*I*)
*R*_int_ = 0.047

#### Refinement

*R*[*F* ^2^ > 2σ(*F* ^2^)] = 0.034
*wR*(*F* ^2^) = 0.083
*S* = 1.04
6922 reflections
196 parameters
H-atom parameters constrained
Δρ_max_ = 0.52 e Å^−3^
Δρ_min_ = −0.43 e Å^−3^

### 

Data collection: *APEX2* (Bruker, 2005 ▶); cell refinement: *APEX2*; data reduction: *SAINT* (Bruker, 2005 ▶); program(s) used to solve structure: *SHELXTL* (Sheldrick, 2008 ▶); program(s) used to refine structure: *SHELXTL*; molecular graphics: *SHELXTL*; software used to prepare material for publication: *SHELXTL* and *PLATON* (Spek, 2003 ▶).

## Supplementary Material

Crystal structure: contains datablocks global, I. DOI: 10.1107/S1600536808002262/ci2558sup1.cif

Structure factors: contains datablocks I. DOI: 10.1107/S1600536808002262/ci2558Isup2.hkl

Additional supplementary materials:  crystallographic information; 3D view; checkCIF report

## Figures and Tables

**Table:** 

Cu1—N1	2.0663 (10)
Cu1—S1	2.2649 (3)

**Table:** 

N1^i^—Cu1—N1	104.29 (5)
N1—Cu1—S1^i^	86.94 (3)
N1—Cu1—S1	121.90 (3)
S1^i^—Cu1—S1	134.452 (19)

Symmetry code: (i) 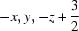.

**Table 2:** Hydrogen-bond geometry (Å, °) *Cg*1 is the centroid of the C1–C6 phenyl ring.

*D*—H⋯*A*	*D*—H	H⋯*A*	*D*⋯*A*	*D*—H⋯*A*
C13—H13*A*⋯S2^ii^	0.93	2.76	3.6698 (15)	167
C11—H11*A*⋯*Cg*1^iii^	0.97	2.98	3.5806 (14)	121

Symmetry codes: (ii) 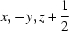; (iii) 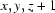.

**Fig. 1.**
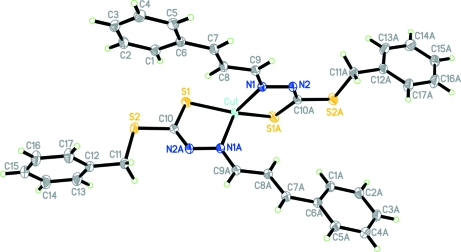
The molecular structure of the title compound, showing 50% probability displacement ellipsoids. Atoms labelled with the suffix A are generated by the symmetry operation (-x, y, 3/2 - z).

**Fig. 2.**
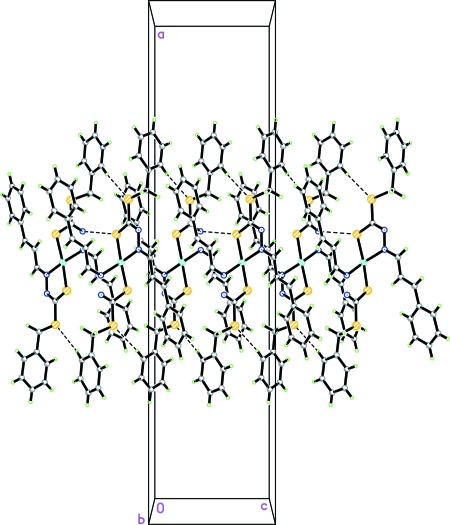
Part of the crystal packing of the title compound, viewed along the b axis. Intermolecular C—H···S weak interactions are shown as dashed lines.

## supplementary crystallographic information

### Comment

Synthesis (Ali & Tarafder, 1977) and crystal structure (Shanmuga Sundara Raj *et al.*, 2000) of *S*-benzyldithiocarbazate (SBDTC) have been reported. We have been greatly involved in the chemistry of Schiff bases derived from SBDTC, and also on their metal complexes because of their interesting physico-chemical properties and potentially useful biological activities (Ali *et al.*, 2002, 2008; Tarafder *et al.*, 2001, 2002). In continuation of our interests, we report herein the syntheses of the cinnamaldehyde Schiff base of SBDTC and its copper complex, along with the *x*-ray structural analysis of the four-coordinated Cu^II^ complex.

The Cu^II^ atom of the title complex, lies on a twofold rotation axis and the asymmetric unit therefore contains one-half of a molecule (Fig. 1). Based on other thiosemicarbazones (Ali *et al.*, 2002; Tarafder *et al.*, 2001, 2002), the coordination mode of the Cu^II^ complex is as expected, i.e bis-chelated through the two azomethine nitrogen atoms and the two thiolate sulfur atoms. The Cu^II^ center is in a distorted tetrahedral geometry with the N_2_S_2_ donor atoms of the two Schiff base ligands (Fig. 1). Both nitrogen atoms (N1 and N1A) and sulfur atoms (S1 and S1A) from the two ligands are coordinated at opposite positions. The N—Cu—N and S—Cu—S bond angles are 104.29 (5)° and 134.452 (14)°, respectively, and reflective of the elongation of the Cu—S bond length [*ca* 0.19 Å] over the Cu—N bond length. The Cu1—N1 and Cu1—S1 distances of 2.0663 (10) Å and 2.2648 (3) Å, respectively, are in the same range as those in other four coordination Cu^II^ complexes of the related Schiff base ligands (Ali *et al.*, 2008; Castiñeiras *et al.*, 1998; Goswami & Eichhorn, 2000). The Cu^II^-bidentate rings are slightly non-planar. The Cu1—S1—N1A—N2A—C10 ring has a maximum deviation of 0.085 (1) Å for the N1A atom. The mean plane of the propenyl moiety (C7/C8/C9) makes a dihedral angle of 12.15 (9)° with mean plane of the attached C1–C6 benzene ring. The dihedral angle between the C1–C6 and C12–C17 phenyl rings of the two ligands is 8.73 (7)°. Bond lengths and angles observed in the Schiff base ligand are of normal values (Allen *et al.*, 1987).

In the crystal packing (Fig. 2), the molecules are interconnected by weak C—H···S intermolecular interactions (Table 1) into chains along the *c* axis. The crystal structure is further stabilized by C—H···π interactions (Table 2) involving the C1—C6 benzene ring (centroid *Cg*1).

### Experimental

The Schiff base ligand was prepared by adding cinamaldehyde (1.32 g, 10 mmol) to a hot solution of *S*-benzyldithiocarbazate (SBDTC) (1.98 g, 10 mmol) in absolute ethanol (40 ml), as reported previously (Ali & Tarafder, 1977). The mixture was refluxed for 10 min. The yellow precipitate which formed was isolated and washed with hot ethanol. The yellow solid product was recrystallized from absolute ethanol (yield: 1.52 g, 46%). The copper complex was synthesized by adding the copper nitrate trihydrate (0.31 g, 0.5 mmol) in ethanol (10 ml) to a hot solution of the above Schiff base ligand (0.31 g, 1 mmol) in ethanol (80 ml) and the reaction mixture was refluxed for 5 min when a brownish precipitate was formed. The product was separated and washed with hot ethanol (yield: 0.32 g, 74%). Green single crystals of the title complex were recrystallized from a chloroform-absolute ethanol (10:3 V/V) solution after 20 d at room temperature.

### Refinement

All H atoms were positioned geometrically and allowed to ride on their parent atoms, with C—H distances in the range 0.93–0.97 Å. The *U*_iso_ values were constrained to be 1.2*U*_eq_ of the carrier atom. The highest residual density peak is located 0.38 Å from Cu1 and the deepest hole is located 0.46 Å from S2.

### Crystal data

**Table:**

[Cu(C_17_H_15_N_2_S_2_)_2_]	*F*_000_ = 1420
*M**_r_* = 686.45	*D*_x_ = 1.449 Mg m^−^^3^
Orthorhombic, *P**b**c**n*	Mo *K*α radiation λ = 0.71073 Å
Hall symbol: -P 2n 2ab	Cell parameters from 6922 reflections
*a* = 36.1410 (7) Å	θ = 2.1–35.0º
*b* = 9.9372 (2) Å	µ = 0.99 mm^−^^1^
*c* = 8.7598 (2) Å	*T* = 100.0 (1) K
*V* = 3146.00 (11) Å^3^	Plate, green
*Z* = 4	0.57 × 0.29 × 0.10 mm

### Data collection

**Table:**

Bruker SMART APEXII CCD area-detector diffractometer	6922 independent reflections
Radiation source: fine-focus sealed tube	5675 reflections with *I* > 2σ(*I*)
Monochromator: graphite	*R*_int_ = 0.047
Detector resolution: 8.33 pixels mm^-1^	θ_max_ = 35.0º
*T* = 100.0(1) K	θ_min_ = 2.1º
ω scans	*h* = −58→57
Absorption correction: multi-scan(SADABS; Bruker, 2005)	*k* = −16→16
*T*_min_ = 0.603, *T*_max_ = 0.906	*l* = −13→14
84850 measured reflections	

### Refinement

**Table:**

Refinement on *F*^2^	Secondary atom site location: difference Fourier map
Least-squares matrix: full	Hydrogen site location: inferred from neighbouring sites
*R*[*F*^2^ > 2σ(*F*^2^)] = 0.034	H-atom parameters constrained
*wR*(*F*^2^) = 0.083	*w* = 1/[σ^2^(*F*_o_^2^) + (0.0303*P*)^2^ + 2.083*P*] where *P* = (*F*_o_^2^ + 2*F*_c_^2^)/3
*S* = 1.04	(Δ/σ)_max_ = 0.001
6922 reflections	Δρ_max_ = 0.52 e Å^−^^3^
196 parameters	Δρ_min_ = −0.43 e Å^−^^3^
Primary atom site location: structure-invariant direct methods	Extinction correction: none

### Special details

**Table:**

Experimental. The low-temparture data was collected with the Oxford Cyrosystem Cobra low-temperature attachment.
Geometry. All e.s.d.'s (except the e.s.d. in the dihedral angle between two l.s. planes) are estimated using the full covariance matrix. The cell e.s.d.'s are taken into account individually in the estimation of e.s.d.'s in distances, angles and torsion angles; correlations between e.s.d.'s in cell parameters are only used when they are defined by crystal symmetry. An approximate (isotropic) treatment of cell e.s.d.'s is used for estimating e.s.d.'s involving l.s. planes.
Refinement. Refinement of *F*^2^ against ALL reflections. The weighted *R*-factor *wR* and goodness of fit *S* are based on *F*^2^, conventional *R*-factors *R* are based on *F*, with *F* set to zero for negative *F*^2^. The threshold expression of *F*^2^ > σ(*F*^2^) is used only for calculating *R*-factors(gt) *etc*. and is not relevant to the choice of reflections for refinement. *R*-factors based on *F*^2^ are statistically about twice as large as those based on *F*, and *R*- factors based on ALL data will be even larger.

### Fractional atomic coordinates and isotropic or equivalent isotropic displacement parameters (Å^2^)

**Table:**

	*x*	*y*	*z*	*U*_iso_*/*U*_eq_	
Cu1	0.0000	0.07778 (2)	0.7500	0.01578 (5)	
S1	−0.056798 (8)	−0.01045 (3)	0.70614 (4)	0.02194 (6)	
S2	−0.126264 (8)	0.09538 (4)	0.81392 (4)	0.02454 (7)	
N1	0.02535 (3)	0.20538 (10)	0.59588 (11)	0.01718 (17)	
N2	0.06393 (3)	0.20720 (10)	0.59855 (12)	0.01796 (17)	
C1	−0.10931 (3)	0.28518 (12)	0.39861 (14)	0.0206 (2)	
H1A	−0.1006	0.2148	0.4586	0.025*	
C2	−0.14653 (3)	0.29131 (13)	0.36079 (15)	0.0229 (2)	
H2A	−0.1626	0.2252	0.3961	0.027*	
C3	−0.15998 (3)	0.39565 (14)	0.27034 (15)	0.0239 (2)	
H3A	−0.1850	0.3998	0.2462	0.029*	
C4	−0.13588 (4)	0.49338 (15)	0.21640 (16)	0.0251 (2)	
H4A	−0.1447	0.5625	0.1548	0.030*	
C5	−0.09847 (3)	0.48806 (13)	0.25450 (15)	0.0222 (2)	
H5A	−0.0825	0.5539	0.2181	0.027*	
C6	−0.08476 (3)	0.38468 (12)	0.34685 (13)	0.01828 (19)	
C7	−0.04535 (3)	0.38225 (12)	0.38369 (14)	0.0192 (2)	
H7A	−0.0305	0.4468	0.3374	0.023*	
C8	−0.02862 (3)	0.29470 (12)	0.47880 (14)	0.0194 (2)	
H8A	−0.0433	0.2341	0.5325	0.023*	
C9	0.01065 (3)	0.29035 (12)	0.50134 (13)	0.01849 (19)	
H9A	0.0258	0.3493	0.4474	0.022*	
C10	−0.07825 (3)	0.11156 (12)	0.81865 (13)	0.01794 (19)	
C11	−0.14240 (3)	0.20513 (14)	0.96662 (15)	0.0230 (2)	
H11A	−0.1295	0.1852	1.0611	0.028*	
H11B	−0.1381	0.2987	0.9405	0.028*	
C12	−0.18327 (3)	0.17791 (13)	0.98351 (14)	0.0210 (2)	
C13	−0.19556 (4)	0.07096 (16)	1.07276 (17)	0.0295 (3)	
H13A	−0.1784	0.0166	1.1223	0.035*	
C14	−0.23304 (4)	0.04438 (17)	1.08878 (18)	0.0326 (3)	
H14A	−0.2409	−0.0266	1.1499	0.039*	
C15	−0.25879 (3)	0.12346 (16)	1.01385 (16)	0.0282 (3)	
H15A	−0.2839	0.1059	1.0246	0.034*	
C16	−0.24696 (4)	0.22864 (16)	0.92307 (19)	0.0318 (3)	
H16A	−0.2642	0.2813	0.8717	0.038*	
C17	−0.20935 (4)	0.25621 (15)	0.90804 (18)	0.0284 (3)	
H17A	−0.2016	0.3275	0.8471	0.034*	

### Atomic displacement parameters (Å^2^)

**Table:**

	*U*^11^	*U*^22^	*U*^33^	*U*^12^	*U*^13^	*U*^23^
Cu1	0.01427 (8)	0.01721 (9)	0.01586 (8)	0.000	0.00375 (6)	0.000
S1	0.02087 (12)	0.02213 (13)	0.02284 (13)	−0.00456 (10)	0.00520 (10)	−0.00534 (10)
S2	0.01540 (12)	0.03281 (16)	0.02541 (15)	−0.00349 (11)	−0.00031 (10)	−0.00859 (12)
N1	0.0146 (4)	0.0197 (4)	0.0172 (4)	0.0005 (3)	0.0006 (3)	−0.0007 (3)
N2	0.0132 (4)	0.0216 (4)	0.0190 (4)	−0.0009 (3)	0.0002 (3)	0.0003 (3)
C1	0.0179 (5)	0.0216 (5)	0.0222 (5)	0.0011 (4)	0.0005 (4)	0.0008 (4)
C2	0.0181 (5)	0.0250 (5)	0.0254 (6)	−0.0006 (4)	0.0010 (4)	−0.0017 (5)
C3	0.0180 (5)	0.0302 (6)	0.0236 (6)	0.0046 (4)	−0.0021 (4)	−0.0034 (5)
C4	0.0225 (5)	0.0287 (6)	0.0242 (6)	0.0055 (5)	−0.0033 (4)	0.0034 (5)
C5	0.0213 (5)	0.0231 (5)	0.0222 (5)	0.0013 (4)	−0.0004 (4)	0.0031 (4)
C6	0.0165 (4)	0.0205 (5)	0.0178 (5)	0.0017 (4)	−0.0001 (4)	−0.0009 (4)
C7	0.0169 (4)	0.0209 (5)	0.0199 (5)	−0.0002 (4)	0.0008 (4)	0.0006 (4)
C8	0.0153 (4)	0.0224 (5)	0.0203 (5)	−0.0004 (4)	0.0006 (4)	0.0016 (4)
C9	0.0161 (4)	0.0213 (5)	0.0181 (5)	−0.0003 (4)	0.0005 (4)	0.0009 (4)
C10	0.0159 (4)	0.0213 (5)	0.0167 (5)	−0.0014 (4)	0.0009 (3)	0.0001 (4)
C11	0.0153 (5)	0.0284 (6)	0.0254 (6)	−0.0008 (4)	−0.0003 (4)	−0.0058 (5)
C12	0.0149 (4)	0.0257 (5)	0.0223 (5)	0.0001 (4)	−0.0014 (4)	−0.0032 (4)
C13	0.0198 (5)	0.0381 (7)	0.0308 (7)	0.0006 (5)	−0.0043 (5)	0.0095 (6)
C14	0.0224 (6)	0.0428 (8)	0.0326 (7)	−0.0063 (5)	−0.0008 (5)	0.0105 (6)
C15	0.0159 (5)	0.0398 (7)	0.0290 (6)	−0.0030 (5)	0.0005 (4)	−0.0015 (6)
C16	0.0171 (5)	0.0363 (7)	0.0419 (8)	0.0034 (5)	−0.0039 (5)	0.0057 (6)
C17	0.0190 (5)	0.0294 (6)	0.0370 (7)	0.0005 (5)	−0.0012 (5)	0.0070 (5)

### Geometric parameters (Å, °)

**Table:**

Cu1—N1^i^	2.0663 (10)	C6—C7	1.4606 (16)
Cu1—N1	2.0663 (10)	C7—C8	1.3478 (17)
Cu1—S1^i^	2.2648 (3)	C7—H7A	0.93
Cu1—S1	2.2649 (3)	C8—C9	1.4335 (16)
S1—C10	1.7442 (12)	C8—H8A	0.93
S2—C10	1.7432 (11)	C9—H9A	0.93
S2—C11	1.8217 (13)	C10—N2^i^	1.3027 (15)
N1—C9	1.2965 (15)	C11—C12	1.5088 (16)
N1—N2	1.3949 (13)	C11—H11A	0.97
N2—C10^i^	1.3027 (15)	C11—H11B	0.97
C1—C2	1.3866 (16)	C12—C17	1.3897 (18)
C1—C6	1.4037 (17)	C12—C13	1.3922 (19)
C1—H1A	0.93	C13—C14	1.3871 (19)
C2—C3	1.3926 (19)	C13—H13A	0.93
C2—H2A	0.93	C14—C15	1.384 (2)
C3—C4	1.388 (2)	C14—H14A	0.93
C3—H3A	0.93	C15—C16	1.381 (2)
C4—C5	1.3937 (17)	C15—H15A	0.93
C4—H4A	0.93	C16—C17	1.3927 (19)
C5—C6	1.3983 (17)	C16—H16A	0.93
C5—H5A	0.93	C17—H17A	0.93
			
N1^i^—Cu1—N1	104.29 (5)	C7—C8—C9	123.27 (11)
N1^i^—Cu1—S1^i^	121.90 (3)	C7—C8—H8A	118.4
N1—Cu1—S1^i^	86.94 (3)	C9—C8—H8A	118.4
N1^i^—Cu1—S1	86.94 (3)	N1—C9—C8	120.88 (11)
N1—Cu1—S1	121.90 (3)	N1—C9—H9A	119.6
S1^i^—Cu1—S1	134.452 (19)	C8—C9—H9A	119.6
C10—S1—Cu1	92.17 (4)	N2^i^—C10—S2	118.41 (9)
C10—S2—C11	104.25 (6)	N2^i^—C10—S1	130.18 (9)
C9—N1—N2	114.32 (10)	S2—C10—S1	111.41 (6)
C9—N1—Cu1	129.45 (8)	C12—C11—S2	106.15 (8)
N2—N1—Cu1	116.12 (7)	C12—C11—H11A	110.5
C10^i^—N2—N1	113.39 (10)	S2—C11—H11A	110.5
C2—C1—C6	120.33 (11)	C12—C11—H11B	110.5
C2—C1—H1A	119.8	S2—C11—H11B	110.5
C6—C1—H1A	119.8	H11A—C11—H11B	108.7
C1—C2—C3	120.49 (12)	C17—C12—C13	118.55 (11)
C1—C2—H2A	119.8	C17—C12—C11	121.14 (12)
C3—C2—H2A	119.8	C13—C12—C11	120.29 (11)
C4—C3—C2	119.71 (11)	C14—C13—C12	120.92 (13)
C4—C3—H3A	120.1	C14—C13—H13A	119.5
C2—C3—H3A	120.1	C12—C13—H13A	119.5
C3—C4—C5	120.07 (12)	C15—C14—C13	120.04 (14)
C3—C4—H4A	120.0	C15—C14—H14A	120.0
C5—C4—H4A	120.0	C13—C14—H14A	120.0
C4—C5—C6	120.68 (12)	C16—C15—C14	119.66 (12)
C4—C5—H5A	119.7	C16—C15—H15A	120.2
C6—C5—H5A	119.7	C14—C15—H15A	120.2
C5—C6—C1	118.71 (11)	C15—C16—C17	120.35 (13)
C5—C6—C7	119.04 (11)	C15—C16—H16A	119.8
C1—C6—C7	122.23 (11)	C17—C16—H16A	119.8
C8—C7—C6	125.77 (11)	C12—C17—C16	120.46 (13)
C8—C7—H7A	117.1	C12—C17—H17A	119.8
C6—C7—H7A	117.1	C16—C17—H17A	119.8
			
N1^i^—Cu1—S1—C10	6.84 (5)	C1—C6—C7—C8	−5.87 (19)
N1—Cu1—S1—C10	−98.11 (5)	C6—C7—C8—C9	174.88 (11)
S1^i^—Cu1—S1—C10	140.38 (4)	N2—N1—C9—C8	176.16 (10)
N1^i^—Cu1—N1—C9	−64.62 (10)	Cu1—N1—C9—C8	−7.90 (17)
S1^i^—Cu1—N1—C9	173.21 (11)	C7—C8—C9—N1	179.00 (12)
S1—Cu1—N1—C9	30.76 (12)	C11—S2—C10—N2^i^	−11.46 (12)
N1^i^—Cu1—N1—N2	111.27 (8)	C11—S2—C10—S1	168.25 (7)
S1^i^—Cu1—N1—N2	−10.91 (7)	Cu1—S1—C10—N2^i^	−4.35 (12)
S1—Cu1—N1—N2	−153.36 (7)	Cu1—S1—C10—S2	175.98 (6)
C9—N1—N2—C10^i^	−172.92 (11)	C10—S2—C11—C12	−171.27 (9)
Cu1—N1—N2—C10^i^	10.56 (12)	S2—C11—C12—C17	−94.14 (13)
C6—C1—C2—C3	0.38 (19)	S2—C11—C12—C13	84.28 (14)
C1—C2—C3—C4	0.7 (2)	C17—C12—C13—C14	−1.3 (2)
C2—C3—C4—C5	−0.9 (2)	C11—C12—C13—C14	−179.71 (14)
C3—C4—C5—C6	0.1 (2)	C12—C13—C14—C15	0.9 (2)
C4—C5—C6—C1	0.94 (19)	C13—C14—C15—C16	0.1 (2)
C4—C5—C6—C7	179.73 (12)	C14—C15—C16—C17	−0.7 (2)
C2—C1—C6—C5	−1.18 (18)	C13—C12—C17—C16	0.6 (2)
C2—C1—C6—C7	−179.93 (11)	C11—C12—C17—C16	179.09 (13)
C5—C6—C7—C8	175.39 (12)	C15—C16—C17—C12	0.3 (2)

Symmetry codes: (i) −*x*, *y*, −*z*+3/2.

### Hydrogen-bond geometry (Å, °)

**Table:**

*D*—H···*A*	*D*—H	H···*A*	*D*···*A*	*D*—H···*A*
C13—H13A···S2^ii^	0.93	2.76	3.6698 (15)	167
C11—H11A···Cg1^iii^	0.97	2.98	3.5806 (14)	121

Symmetry codes: (ii) *x*, −*y*, *z*+1/2; (iii) *x*, *y*, *z*+1.
